# Upregulation of MiR-155 in Nasopharyngeal Carcinoma is Partly Driven by LMP1 and LMP2A and Downregulates a Negative Prognostic Marker JMJD1A

**DOI:** 10.1371/journal.pone.0019137

**Published:** 2011-04-26

**Authors:** Zi-Ming Du, Li-Fu Hu, Hai-Yun Wang, Li-Xu Yan, Yi-Xin Zeng, Jian-Yong Shao, Ingemar Ernberg

**Affiliations:** 1 State Key Laboratory of Oncology in South China, and Department of Pathology, Sun Yat-sen University Cancer Center, Guangzhou, P.R. China; 2 Department of Microbiology, Tumor and Cell Biology, Karolinska Institutet, Stockholm, Sweden; Karolinska Institutet, Sweden

## Abstract

The role of microRNA-155 (miR-155) has been associated with oncogenesis of several human tumors. However the expression pattern of miR-155 has not been investigated in nasopharyngeal carcinoma (NPC). The present study was to assess miR-155 expression pattern and its possible function in NPC, to identify its targets and evaluate their clinical applications in NPC. MiR-155 was found to be upregulated in two Epstein-Barr virus (EBV) negative NPC derived cell lines CNE1 and TW03, as well as in NPC clinical samples by quantitative Real-time PCR and *in situ* hybridization detection. EBV encoded LMP1 and LMP2A could further enhance the expression of miR-155 in NPC CNE1 and TW03 cells. JMJD1A and BACH1 were identified as putative targets of miR-155 in a bioinformatics screen. Overexpression of miR-155 downregulated a luciferase transcript fused to the 3′UTR of JMJD1A and BACH1. MiR-155 mimic could downregulate the expression of JMJD1A and BACH1, while miR-155 inhibitor could upregulate JMJD1A expression in NPC cell lines. Moreover, downregulation of JMJD1A was significantly correlated with N stage in TNM classification (*p* = 0.023), a lower five-year survival rate (*p* = 0.021), and a lower five-year disease-free survival rate (*p* = 0.049) of NPC patients. Taken together, up-regulation of miR-155 in NPC is partly driven by LMP1 and LMP2A, and results in downregulation of JMJD1A, which is associated with N stage and poor prognosis of NPC patients. The potential of miR-155 and JMJD1A as therapeutic targets in NPC should be further investigated.

## Introduction

Nasopharyngeal carcinoma (NPC) is one of the most common malignancies in certain areas of South-China, Southeast-Asia and North Africa [1]. Epstein-Barr virus (EBV) infection, genetic alterations and other environmental factors have been reported to be associated with risk for NPC [2], [3]. NPC has a dominant clinicopathological behavior of loco-regional recurrence and metastasis, which differs from other types of head and neck cancers [4]. Although NPC tumors are sensitive to radiotherapy and chemotherapy, treatment failure is high due to regional lymph node metastasis, distant metastasis and local recurrence [5]. However, the pathogenesis of NPC is still unclear.

MicroRNAs are an abundant class of non-coding RNAs, typically 20–23 nucleotides in length, which often are evolutionarily conserved in metazoans and expressed in a cell and tissue specific manner. MicroRNAs exert their gene regulatory activity primarily by imperfect base pairing to the 3′ UTR of their target mRNAs, leading to mRNA degradation or translational inhibition. They are involved in numerous cellular processes including proliferation, differentiation, apoptosis and metabolism [6]. MicroRNA-155 (MiR-155) is a microRNA involved in different biological processes including haematopoiesis, inflammation and immunity. Deregulation of miR-155 has been found to be associated with different kinds of cancer, cardiovascular diseases and viral infections [7]. EBV can induce miR-155 expression in B cells resulting in modulation of EBV-regulated gene expression, including attenuation of NF-kappaB signaling [8], [9], [10].

Jumonji Domain 1A (JMJD1A), which is also known as KDM3A (lysine (K)-specific demethylase 3A) plays a role in stem cell differentiation and spermatogenesis and as a cofactor of the androgen receptor and is upregulated by HIF-1 (Hypoxia-inducible factor 1) in hypoxia [11], [12], [13]. BTB and CNC homology 1 (BACH1) is a transcription factor that belongs to the cap'n'collar type of basic region leucine zipper factor family (CNC-bZip) [14]. BACH1 is a recognized hypoxic regulator and functions as an inducible repressor for the HO-1 (Heme oxygenase 1) gene in several human cell types [15]. In addition, BACH1 has been identified as miR-155's direct target in many studies [8], [16], [17].

Due to its proposed role in cancer and its dependence on EBV, we assessed miR-155 expression pattern in NPC, identified its direct targets and evaluated their clinical application in NPC. Here we provide evidence that miR155 is upregulated in NPC, further enhanced by EBV encoded latent membrane protein 1 (LMP1) and latent membrane protein 2A (LMP2A). This results in downregulation of JMJD1A, which is associated with N stage and poor prognosis of NPC patients.

## Results

### MiR-155 is upregulated in NPC

**Figure 1:**
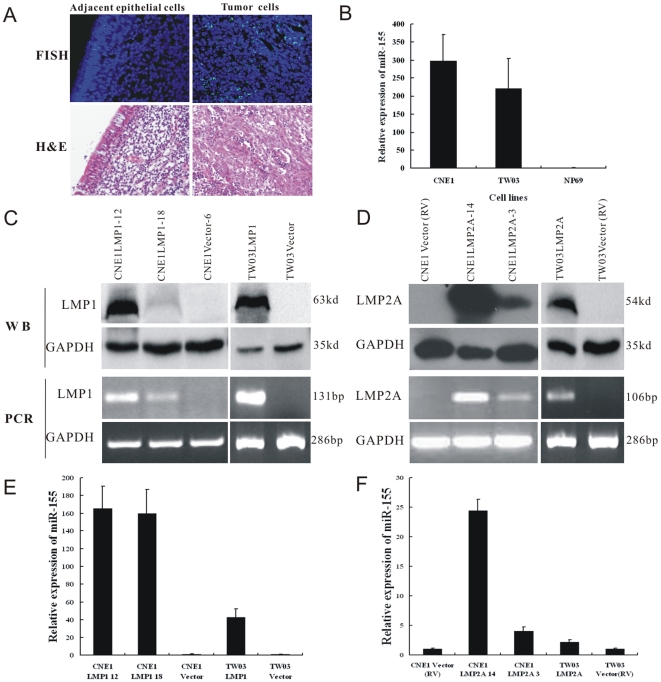
miR-155 was upregulated in NPC and further enhanced by LMP1 and LMP2A. (A) Upregulation of miR-155 in NPC tumor cells, compared with the adjacent epithelial cells. (B) miR-155 was overexpressed in two EBV negative NPC derived cell lines CNE1 (298.5±70.8-fold) and TW03 (222.3±80.6-fold), compared with NP69 cells. (**C**). The expression level of LMP1 checked by PCR and Western Blot Assay in LMP1 stable transfected CNE1 and TW03 cells respectively. (D). The expression level of LMP2A checked by PCR and Western Blot Assay in LMP2A stable transfected CNE1 and TW03 cells respectively. Then qPCR was performed to detect miR-155 expression. Overexpression of miR-155 was found in LMP1 (E) and LMP2A (F) stable transfected CNE1 and TW03 cells respectively.

In situ hybridization was performed to evaluate miR-155 expression in NPC tumor cells and normal nasopharyngeal epithelium. Strong expression of miR-155 was observed in NPC tumor cells, while weak expression was observed in normal adjacent nasopharyngeal epithelium (**Fig. 1A**). MiR-155 expression was also significantly upregulated in two EBV negative NPC-derived cell lines CNE1 and TW03, compared to NP69 cells from normal nasopharyngeal epithelium (**Fig. 1B**).

### EBV LMP1 and LMP2A further enhance miR-155 expression in NPC

In order to investigate whether EBV encoded LMP1 and LMP2A could influence miRNAs expression in NPC, miRNAs microarray was employed to analyze the differential miRNAs induced by LMP1 and LMP2A in NPC TW03 cells. We found that LMP1 could induce the expression of several miRNAs such as miR-155, miR-188, miR-181b while other cellular miRNAs such as miR-103, miR-107 were downregulated. LMP2A also induced the expression of a variety of cellular miRNAs such as miR-155, miR-188, miR-181b while some other cellular miRNAs such as miR-125b were downregulated (**Table 1**).

**Table 1:** The differential miRNAs induced by LMP1 and LMP2A in NPC TW03 cells with miRNAs microarray screening.

TWO3-LMP1 *vs* TW03				TWO3-LMP2A *vs* TW03			
Gene Name	F.C.	Score	Reg.	Gene Name	F.C.	Score	Reg.
hsa-miR-155	8.36	15.61	Up	hsa-miR-155	5.02	10.47	Up
hsa-miR-188	10.28	7.79	Up	hsa-miR-188	7.25	6.40	Up
hsa-miR-181b	4.88	10.04	Up	hsa-miR-181b	3.66	4.74	Up
hsa-miR-361	3.08	7.99	Up	hsa-miR-361	2.34	5.10	Up
hsa-miR-134	100.98	9.66	Up	hsa-miR-93	2.41	7.50	Up
hsa-miR-516-3p	22.80	12.34	Up	hsa-miR-18a	2.10	4.78	Up
hsa-miR-520b	29.77	6.13	Up	hsa-miR-200c	0.47	−6.30	Down
hsa-miR-520e	42.07	14.35	Up	hsa-miR-125b	0.23	−5.23	Down
hsa-miR-202	6.36	11.81	Up				
hsa-miR-365	3.83	12.99	Up				
hsa-miR-28	2.03	7.82	Up				
hsa-miR-200a	0.40	−5.37	Down				
hsa-miR-107	0.27	−12.20	Down				
hsa-miR-103	0.18	−11.21	Down				
hsa-miR-15b	0.35	−11.30	Down				

SAM was used for data analysis. F.C.: Fold Change; Reg.: Regulation.

The miRNA microarray was next validated by qPCR. According to our miRNA microarray data, we chose miR-155, which was upregulated by LMP1 and LMP2A in TW03 cells; miR-200c, which was downregulated by LMP1 and LMP2A in TW03 cells, and miR-146a, which was unaffected by LMP1 and LMP2A transfection of TW03 cells. The miRNA array data and qPCR results correlated well (**Fig S1**).

Moreover, two CNE1LMP1 stable transfected clones (CNE1LMP1-12 and CNE1LMP1-18), one TW03LMP1 stable transfected clone (TW03LMP1), two CNE1LMP2A stable transfected clones (CNE1LMP2A-14 and CNE1LMP2A-3) and one TW03LMP2A stable transfected clone (TW03LMP2A) were used to validate the role of LMP1 and LMP2A on miR-155 (**Fig. 1C and 1D**). Compared with vector control, miR-155 expression was increased in two CNE1LMP1 clones and in one TW03LMP1 clone (**Fig. 1E**). LMP2A induced miR-155 expression in two CNE1LMP2A clones and in one TW03LMP2A clone (**Fig. 1F**).

### The prediction of putative targets of miR-155

**Figure 2:**
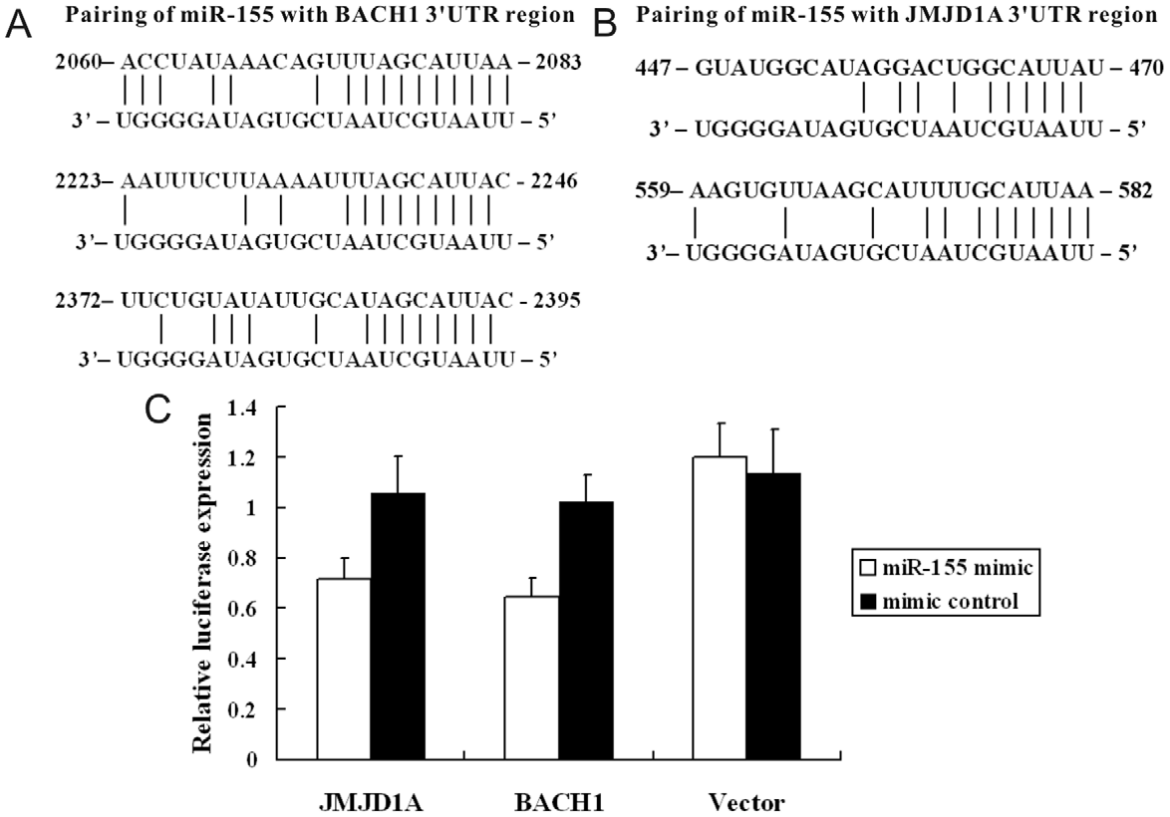
Both JMJD1A and BACH1 were the direct targets of miR-155. (A). Pairing of miR-155 with BACH1 3′UTR region. (B). Pairing of miR-155 with JMJD1A 3′UTR region. (C). Overexpression of miR-155 by the miR-155 mimic resulted in a significant decrease in luciferase signals of pMIR-report-JMJD1A 3′UTR (JMJD1A) and pMIR-report-BACH1 3′UTR (BACH1) transfected HEK 293 cells, but not in pMIR-report-vector (vector) transfected HEK 293 cells.

Four algorithms, miRanda (http://www.microrna.org/miranda.html) [18], TargetScan (http://genes.mit.edu/targetscan) [19], PicTar (http://pictar.bio.nyu.edu) [20] and miRBase (http://microrna.sanger.ac.uk/targets/v2) [21] were used to predict putative targets of miR-155 respectively. Forty-seven common targets which were predicted by at least 3 algorithms were indentified (**Table S1**). Of these, BACH1 (**Fig. 2A**) and JMJD1A (**Fig. 2B**), which have several miR-155 target sites in their 3′UTR region, were selected for further validation.

### JMJD1A and BACH1 are direct target genes of miR-155

To test whether JMJD1A and BACH1 responds to miR-155 through direct 3′UTR interactions, we cloned the 3′UTR of JMJD1A and BACH1 into a reporter plasmid downstream of luciferase. The luciferase reporter assays were performed by transiently transfecting HEK 293T cells respectively, with pMIR-report-JMJD1A 3′UTR, or pMIR-report-BACH1 3′UTR, or pMIR-report-vector (control), together with miR155 mimic (Ambion, USA) or mimic control and pCMV-Renilla (internal control). After 48 hr transfection, a dual-luciferase reporter assay system (Promega, USA) was used to detect luciferase expression. We found that upregulation of miR-155 resulted in downregulation of luciferase fused to the JMJD1A and BACH1 3′UTR in HEK 293T cells. This shows that miR-155 directly targets the JMJD1A and BACH1 3′UTR leading to decreased expression (**Fig. 2C**).

**Figure 3:**
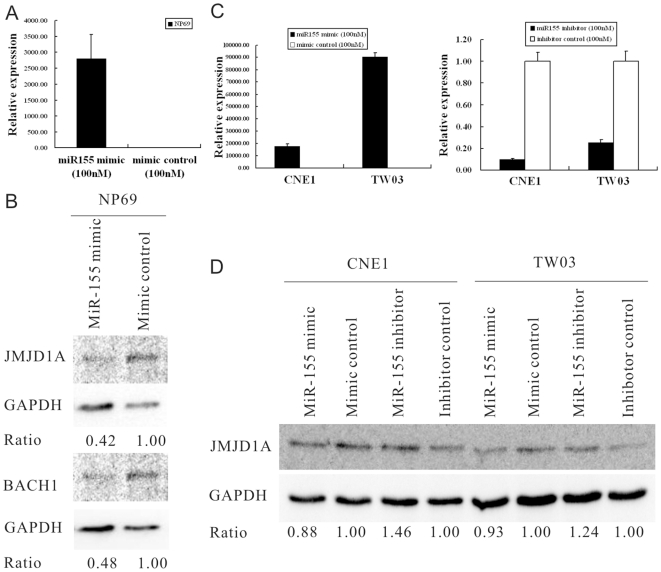
Both JMJD1A and BACH1 expression were regulated by miR-155. (**A**). The relative miR-155 expression in NP69 cells transfected with miR155 mimic (100 nM) to NP69 cells transfected with a negative control (100 nM) by qPCR detection. (B). Immunoblot analysis of JMJD1A and BACH1 expression in cells were analyzed at 48 hr post-transfection, and GAPDH was used as a loading control. MiR-155 mimic could downregulate the expression of JMJD1A and BACH1 in NP69 cells. (C). The relative miR-155 expression in CNE1 and TW03 cells transfected with miR155 mimic (100 nM) or miR155 inhibitor (100 nM) to CNE1 and TW03 cells transfected with a negative control (100 nM) by qPCR detection. (D).Immunoblot analysis of JMJD1A expression in cells was analyzed at 48 hrs post-transfection, and GAPDH was used as a loading control. MiR-155 mimic could downregulate the expression of JMJD1A, while miR-155 inhibitor could upregulate JMJD1A expression in CNE1 and TW03 cells.

To determine whether miR-155 could repress endogenous JMJD1A and BACH1, NP69 cells was transfected with miR155 mimic (100 nM) or with a negative control (100 nM) respectively (**Fig. 3A**). After 48 hr transfection, cells were collected for Western blot assay of JMJD1A and BACH1. Densitometry analysis showed that both JMJD1A and BACH1 expression were decreased by miR155 mimic in NP69 cells (**Fig. 3B**).

For further validation, CNE1 and TW03 cells were transfected with miR155 mimic (100 nM), miR155 inhibitor (100 nM) or a negative control (100 nM) respectively (**Fig. 3C**). After 48 hr transfection, cells were collected for Western blot assay of JMJD1A. Densitometry analysis showed that JMJD1A expression was decreased by miR155 overexpression in CNE1 and TW03 cells, while JMJD1A expression was increased by inhibition of miR155 (**Fig. 3D**).

### JMJD1A and BACH1 are downregulated in NPC

**Figure 4:**
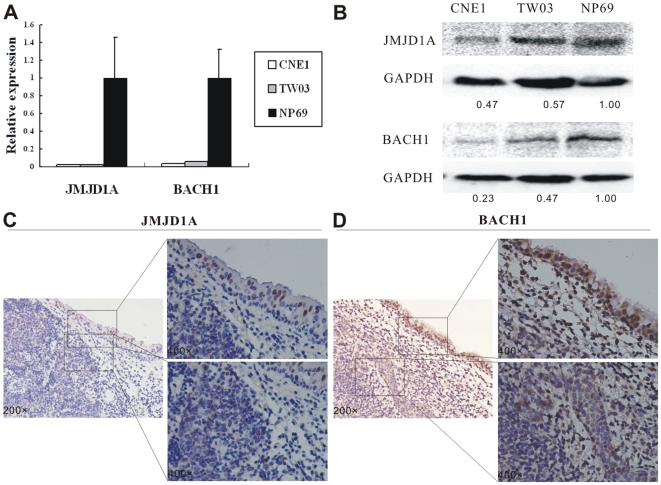
JMJD1A and BACH1 were downregulated in NPC. (A) Q-PCR was performed to detect JMJD1A and BACH1 mRNA expression in NPC CNE1 and TW03 cells, compared to NP69 cells respectively. JMJD1A (CNE1: 0.02±0.002-fold; TW03: 0.02±0.001-fold) and BACH1 (CNE1: 0.03±0.001-fold; TW03: 0.06±0.001-fold) were down-regulated in NPC cells. (B) Western blot demonstrating downregulation of JMJD1A and BACH1 in NPC cells. Downregulation of JMJD1A (C) and BACH1 (D) were found in NPC tumor cells, compared with the adjacent epithelial cells.

To check the expression level of JMJD1A and BACH1 in NPC, qPCR was carried out to detect the expression of JMJD1A and BACH1 mRNA in CNE1, TW03 and NP69 cells. Compared with NP69 cells, the mRNA level of JMJD1A and BACH1 in CNE1, TW03 was significantly lower (**Fig. 4A**). Western blot showed that the protein level of JMJD1A and BACH1 in CNE1, TW03 was also significantly lower, compared to NP69 cells (**Fig. 4B**). Immunostaining was performed to evaluate the expression of JMJD1A and BACH1 in NPC tumor cells and adjacent normal nasopharyngeal epithelium. Weak expression of JMJD1A and BACH1 were observed in nuclear of NPC tumor cells, while strong expression of JMJD1A and BACH1 were observed in normal adjacent nasopharyngeal epithelium (**Fig. 4C and 4D**).

### Downregulation of JMJD1A predicts poor survival in NPC

The expression of JMJD1A and BACH1 was detected by immunostaining in 185 NPC cases. Low expression of JMJD1A was observed in 113 (61.08%), and was associated significantly with N-stage (p = 0.023). No significant association was seen between JMJD1A expression and age, gender, T stage, TNM stage, recurrence or metastasis. In addition, no significant association was seen between BACH1 expression and age, gender, T stage, N stage, TNM stage, recurrence or metastasis (**Table 2**).

**Table 2:** Correlation between JMJD1A and BACH1 expression and clinicopathological parameters of NPC.

Parameters	Cases (n = 185)	JMJD1A expression		*P* value	BACH1 expression		*P* value
		L.E. (n = 113)	H.E. (n = 72)		L.E. (n = 94)	H.E. (n = 91)	
Age							
<47	85	47	38	0.137	37	48	0.068
≥47	100	66	34		57	43	
Gender							
Male	144	88	56	0.987	71	73	0.443
Female	41	25	16		23	18	
WHO classification							
II	160	98	62	0.905	77	83	0.065
III	25	15	10		17	8	
T stage							
T1+T2	73	44	29	0.865	41	32	0.240
T3+T4	112	69	43		53	59	
N stage							
N0	36	16	20	0.023*	19	17	0.793
N1+N2+N3	149	97	52		75	74	
TNM stage							
I+II	47	25	22	0.199	26	21	0.474
III+IV	138	88	50		68	70	
Recurrence							
Yes	11	6	5	0.674	4	7	0.323
No	174	107	67		90	84	
Metastasis							
Yes	19	12	7	0.845	9	10	0.751
No	166	101	65		85	81	

L.E.: Low expression; H.E.: High expression.

**Figure 5:**
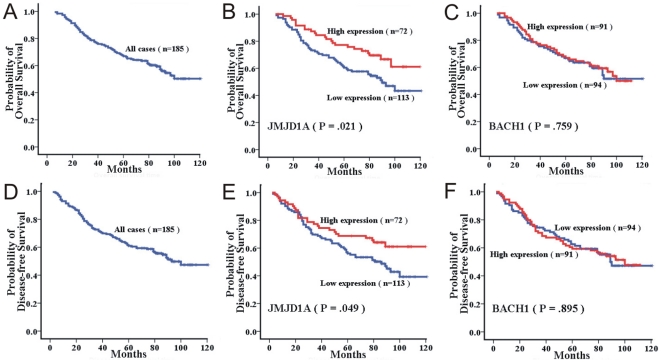
Kaplan-Meier curves for overall survival and disease-free survival of the 185 NPC patients. A, Kaplan-Meier curves for overall survival (OS) of the 185 NPC patients; and B, Kaplan-Meier curves for OS in NPC patients with low level and high level JMJD1A expression; C, Kaplan-Meier curves for OS in NPC patients with low level and high level BACH1 expression;; D, Kaplan-Meier curves for disease-free survival (DFS) of the 185 NPC patients; E, Kaplan-Meier curves for DFS in NPC patients with low level and high level JMJD1A expression; F, Kaplan-Meier curves for DFS in NPC patients with low level and high level BACH1 expression.

Overall survival analysis and disease-free survival analysis was then performed (**Fig. 5A and 5D**). The five-year overall survival rate was 61.3% for patients with low JMJD1A expression (n = 113), and 77.2% for patients with high JMJD1A expression (n = 72), which was a significant difference (p = 0.021, Fig. 5B). The five-year overall survival rate was 66.8% for patients with low BACH1 expression (n = 94), and 68.2% for patients with high BACH1 expression (n = 91), which was no significant difference (p = 0.759, Fig. 5C). Furthermore, the five-year disease-free survival rate was 57.0% for NPC patients with low levels of JMJD1A expression (n = 113), and 68.7% for those with high levels of JMJD1A expression (n = 72), and this difference in the disease-free survival rate was significant (p = 0.049, Fig. 5E). No significant difference was seen in the disease-free survival rate of NPC patients, with or without BACH1 overexpression (p = 0.895, **Fig. 5F**).

## Discussion

MiR-155 is upregulated in several human tumors, such as chronic lymphocytic leukemia [22], melanoma [23], head and neck squamous cell carcinoma [24], clear-cell kidney cancer [25], hepatocellular carcinoma [26], lymphoma [27], [28], [29], [30], [31], thyroid carcinoma [32], breast cancer [33], [34], [35], [36], colon cancer [33], cervical cancer [37], pancreatic cancer [38], [39], [40], and lung cancer [41], [42]. Furthermore, elevated expression of miR-155 was associated with poor prognosis of pancreatic cancer [38] and lung cancer [41], [42]. Recently, Chen et al used a stem-loop real-time-PCR method to quantify the expression levels of 270 human miRNAs in 13 NPC samples and 9 adjacent normal tissues. They identified 35 miRNAs whose expression levels were significantly altered in NPC samples, including upregulation of miR-155 [43]. In the present study, we report that miR-155 is overexpressed in NPC. Hence, our results were consistent with previous studies of miR-155 often being upregulated in other malignancies.

Several studies have demonstrated that EBV could induce miR-155 expression in B cells and cell lines which in turn modulates EBV-regulated pathways [8], [9], [10]. LMP1 [8], [9], [44], [45] and EBNA2 [9] were responsible for the upregulation of miR-155 after EBV infection of B-lymphocytes, while LMP2A did not influence miR-155 expression [8], [45]. Furthermore LMP1 was demonstrated to trans-activate miR-155 transcription through the NF-kappaB and AP1 pathways [8], [45]. In contrast, EBV did not induce the expression of miR155 in HEK 293 and Hela cells [9]. We found that both LMP1 and LMP2A could induce the miR-155 expression in NPC CNE1 and TW03 cells. To our knowledge, this is the first report that LMP2A could induce miR-155 expression in NPC. Guasparri et al reported that EBV LMP2A protein could affect LMP1-mediated NF-κB signaling and survival of lymphoma cells [46], hence LMP2A might increase miR-155 expression through the NF- kappaB pathway.

In addition, LMP1 was reported to induce the expression of miR-146a in B-lymphocytes [44]. However, in our study, we found that neither LMP1 nor LMP2A could induce the miR-146a expression in NPC cells (**Fig S1**). These differences might be due to different tumor types.

LMP1 and LMP2A were expressed in approximately 65% and 45.7% NPC patients, repectively [47], [48], and miR-155 was also found to be upregulated in many human tumors, which were not related to EBV [32], [33], [34], [37], [38], [42]. Furthermore, in our study, both CNE1 and TW03 were EBV negative NPC derived cell lines, and the expression of miR-155 in these two cell lines was still higher than that of the immortalized nasopharyngeal epithelial cell line NP69. Hence there should be some other unknown factors which could also upregulate miR-155 expression in NPC. TGF-beta (Transforming Growth Factor – beta) was verified to induce miR-155 expression and promoter activity through SMAD4 (SMAD family member 5) [49], and elevated serum levels of TGF-beta1 was also found in NPC patients [50]. Hence TGF-beta and SMAD4 pathway might also contribute to miR-155 overexpression in NPC.

At presently, many direct targets of miR-155 have been identified to show oncogenic features of miR-155. MiR-155 promoted the proliferation of breast cancer cells through down-regulation of SOCS1 (Suppressor of cytokine signaling 1) [35] and FOXO3a (Forkhead box O3) [36]. MiR-155 has also been reported to be involved in the development of lymphoma by targeting SMAD5 (SMAD family member 5) [30] and SHIP1 (inositol polyphosphate-5-phosphatase) [51]. MiR-155 could promote pancreatic tumor development through downregulation of TP53INP1 (Tumor protein p53 induced nuclear protein 1) [52]. Moreover, some other genes implicated in differentiation, inflammation and transcriptional regulation, were direct targets of miR-155, including HIF-1 (Hypoxia-inducible factor 1) [16], [53], BACH1 (BTB and CNC homology 1, basic leucine zipper transcription factor1) [8], [16], HIVEP2 (Human immunodeficiency virus type I enhancer binding protein 2) [8], IKKε (Inhibitor of kappa light polypeptide gene enhancer in B cells, kinase) [54], and so on. In our study, we found that miR-155 could repress endogenous JMJD1A and BACH1 protein expression in NP69 cells. Luciferase reporter assay was performed to identify both JMJD1A and BACH1 as direct targets of miR-155 in NPC cells. This is the first report that JMJD1A is a direct target of miR-155. Furthermore, JMJD1A and BACH1 are downregulated in NPC cell lines and NPC tumor tissues.

Hypoxia is a common feature characteristic of many malignancies and promotes biological processes involved in tumor progression. In hypoxia, several genes are involved in erythropoiesis, angiogenesis and cellular energy metabolism, and are activated by a common transcription factor termed hypoxia inducible factor-1 (HIF-1) [55]. The expression of JMJD1A [11] and BACH1 [15] have been reported to be induced by hypoxia. Interestingly, miR-155 could directly downregulate HIF-1 expression [16], [53], BACH1 expression [8], [16] and JMJD1A expression. We therefore suggest that miR-155 is a negative feedback regulator of HIF1α. The effect of hypoxia on miR-155 itself should be considered in future studies. In this study, downregulation of JMJD1A was found to be significantly correlated with N stage, a lower five-year survival rate, and a lower five-year disease-free survival rate of NPC patients. Adam et al. [56] demonstrated that loss of JMJD1A is sufficient to reduce tumor growth of renal cell carcinoma and colon carcinoma in vivo, suggesting that the function of JMJD1A in different cells and tissues depend on cell microenvironment. Hence, the function of JMJD1A and BACH1 in NPC deserve for further study.

In conclusion, upregulation of miR155 in NPC is partly driven by LMP1 and LMP2A. This results in downregaultion of JMJD1A, which is associated with N stage and poor prognosis of NPC patients. The potential of miR-155 and JMJD1A as therapeutic targets for NPC should be further investigated.

## Materials and Methods

### Cell lines, plasmids and tissue samples

Human NPC cell lines CNE1 (EBV negative, from Cancer Center, Sun Yat-sen University, China), TW03 (EBV negative, the generous gift of Prof. Chin-Tarng Lin, National Taiwan University Hospital) [57] and Human Embryonic Kidney 293T cells (from American Tissue Culture Collection, ATCC, Manassas, VA) were cultured in IMEM (Gibco USA) containing 10% fetal calf serum (FCS). The immortalized nasopharyngeal epithelial cell line NP69 (EBV negative, from the University of Hong Kong, China) [58] was cultured in keratinocyte serum-free medium (Invitrogen, Carlsbad, CA) supplemented with 25 µg/ml bovine pituitary extract, and 0.2 ng/ml recombinant epidermal growth factor, as suggested by the manufacturer.

LMP1 expressing vector (pJ124-A8-CAO-LMP1) [59] and LMP2A expressing vector (pLNPOX/HPH6-LMP2A) [60] were used for stable transfection. CNE1 and TW03 cells that stably expressed LMP1 were generated by Mycophenolic acid, Xanthine and Hypoxanthine selection. CNE1 and TW03 cells that stably expressed LMP2A were generated by retrovirus infection and G418 selection. All the cell lines were grown in a humidified incubator at 37°C with 5% CO_2_.

The NPC biopsies with clinical information were obtained from Sun Yat-Sen University Cancer Center (Guangzhou, China). For this retrospective study, archival formalin-fixed, paraffin-embedded (FFPE) tissue specimens from 185 primary NPC patients (144 males and 41 females; aged from 16 to 73 years; median, 47 years) who underwent radical radiotherapy from 1999–2003 were obtained from the Sun Yat-sen University Cancer Center (Guangzhou, China). The study was approved by the Research Ethics Committee of Sun Yat-Sen University Cancer Center, Guangzhou, China (Reference number: YP-2009175) and Karolinska Institutet, Stockholm, Sweden (Reference number: 00-302). All NPC samples in our study were obtained before treatment with standard curative radiotherapy, with or without chemotherapy. The disease stages of all patients were classified or reclassified according to the China 1992 NPC staging system (see **File S1**) [61]. Of the 185 primary NPC patients, 3 were classified as stage I, 44 as stage II, 88 as stage III, and 50 as stage IV.

### MiRNA microarray analyses

Total RNA samples were analyzed by CapitalBio (CapitalBio Corp. Beijing, China) for miRNA microarray. Procedures were performed as described in detail on the website of CapitalBio (http://www.capitalbio.com). Briefly, miRNA was separated from 30–50 mg total RNA using the Ambion miRNA Isolation Kit. Fluorescein-labeled miRNA [62] were used for hybridization on each miRNA microarray chip containing 509 probes in triplicate, corresponding to 435 human (including 122 predicted miRNAs), 261 mouse, and 196 rat miRNAs found in the miRNA Registry (http://microrna.sanger.ac.uk/sequences/; accessed October 2005). Image intensities were measured as a function of the median of foreground minus the background, as previously described [63]. Raw data were normalized and analyzed in GenePix Pro 4.0 software (Axon Instruments). Expression data were median-centered using the global median normalization function of the Bioconductor package (http://www.bioconductor.org). Statistical comparisons were performed with the SAM software (SAM version 2.1, http://www.stat.stanford.edu/ztibs/SAM/index.html) [64]. All microarray data, which were Minimum Information About a Microarray Experiment (MIAME) compliant, have been deposited to the Gene Expression Omnibus public database with accession number GSE26596.

### Prediction miRNAs target analysis

We analyzed the putative targets of miRNAs as follows [65]: Firstly, the analysis was done by using four algorithms, miRanda (http://www.microrna.org/miranda.html) [18], TargetScan (http://genes.mit.edu/targetscan); [19], PicTar (http://pictar.bio.nyu.edu) [20] and miRBase (http://microrna.sanger.ac.uk/targets/v2) [21] respectively. Because any of the four approaches generates an unpredictable number of false positives, results were intersected to identify the genes commonly predicted by at least three of the methods.

### PCR assays

For miRNAs quantitive realtime PCR (qPCR) assay, total RNA from cell lines was isolated using Trizol reagent (Invitrogen) according to the manufacturer's instructions, then was treated with RNase free DNase I (Cat#: 04716728001, Roche). The miR-155 quantitive realtime PCR assay was performed by TaqMan® MicroRNA Assays (Cat#: 4373124, Applied Biosystems, USA) and RNU6B (Cat#: 4373381, Applied Biosystems, USA) was used as internal control. The relative expression level was determined as 2^−ΔΔCt^. Data are presented as the expression level relative to the calibrator (control sample), with the standard error of the mean of triplicate measures for each test sample.

For mRNA quantitive realtime PCR assay, total RNA was extracted from cell lines using TRIzol reagent (Invitrogen). After reverse transcription of the total RNA, the first-strand cDNA was then used as template for detection of JMJD1A, BACH1, expression by quantitative real time PCR (qPCR) with the SYBR Green I chemistry (Power SYBR Green PCR Master Mix, CAT#: 4367659, ABI Inc., USA). GAPDH was used as internal control. The primers were JMJD1A (Forward: GTC AAC TGT GAG GAG ATT CCA GC and Reverse: AAC TTC AAC ATG AAT CAG TGA CGG); BACH1 (Forward: ATT CAT GCT TCT GTT CAG CCA A and Reverse: GGC ACT GAG AAG CAG GAT CTT T); GAPDH (Forward: AGC CAC ATC GCT CAG ACA C and Reverse: GCC CAA TAC GAC CAA ATC C). The relative expression level was determined as 2^−ΔΔCt^. Data are presented as the expression level relative to the calibrator (control sample), with the standard error of the mean of triplicate measures for each test sample.

For normal PCR assay, total RNA was extracted from cell lines using TRIzol reagent (Invitrogen). After reverse transcription of the total RNA, the first-strand cDNA was then used as templates for detection of LMP1 and LMP2A expression. GAPDH was used as internal control. The primers were LMP1 (131 bp, 55°C) (Forward: AGG TTG AAA ACA AAG GAG GTG ACC A and Reverse: GGA ACC AGA AGA ACC CAA AAG CA); LMP2A (106 bp, 60°C) (Forward: TCC CTA GAA ATG GTG CCA ATG and Reverse: GAA GAG CCA GAA GCA GAT GGA T); GAPDH (286 bp, 56°C) (Forward: CCA CCA TGG AGA AGG CTG GGG CTC A and Reverse: ATC ACG CCA CAG TTT CCC GGA GGG G).

### Western blot assays

Cells were harvested and lysed with RIPA buffer (Upstate, USA). Equal amounts of denatured protein sample were separated by SDS-PAGE and were then transferred electrophoretically to PVDF membranes (Pall, USA) for immunoblot analysis. Antibodies used for immunoblot analysis were against JMJD1A (1∶100 dilution, 12835-1-AP, Proteintech Group, Inc, USA), BACH1 (1∶200 dilution, sc-14700, Santa Cruz, USA); LMP1 (1∶500 dilution, S12) [66], LMP2A (1∶1,000 dilution, 14B7, ITN GmbH, Neuherberg, Germany) and an anti-GAPDH antibody (1∶3,000 dilution, sc-32233, Santa Cruz, USA) was used as loading control. All protein bands were detected using an enhanced chemiluminescent (ECL) Western blot Kit (Cell Signaling Technology, USA).

### MiRNA transfections

Before transfection, 2×10^5^ cells per well were plated into 6-well plates and grown for one day in antibiotic-free medium containing 10% FCS. When the cell confluent was reached to 40% to 60%, cells were transfected with miR-155 Pre-miR™ miRNA Precursor Molecules (Cat#: PM12601, Ambion, USA), or Pre-miR™ miRNA Precursor Molecules-Negative Control #1 (Cat#: AM17110, Ambion, USA) or miR-155 Anti-miR™ miRNA Inhibitor (Cat#: AM12601, Ambion, USA), or Anti-miR™ miRNA Inhibitors-Negative Control #1 (Cat#: AM17010, Ambion, USA) using Lipofectamine 2000 (Invitrogen, USA) according to the manufacturer's instructions. Transfected cells were grown at 37°C for 6 hr, followed by incubation with complete medium. For miR-155 assay and Western blot analysis, cells were harvested for RNA and protein respectively after 48 hr.

### Luciferase reporter assays

3′ UTR sequences of JMJD1A and BACH1 containing the putative miR-155 target sites were isolated from TW03 cDNA by PCR and cloned immediately downstream from the luciferase reading frame in the plasmid pMIR-report-Vector (Cat#: AM5795, Ambion, USA). Primers used for PCR were as follows: JMJD1A (Forward: CAG ACT AGT TAA AAG CAA AAC CTC GTA TC and Reverse: CAG AAG CTT TAA TGC AAA ATG CTT AAC AC); BACH1 (Forward: CAG ACT AGT AAG CCA ATG GAA CCC TTG ATT and Revese: CAG AAG CTT GCC TTG AAA CAT TTT CTT AGA A). All inserts were sequenced in their entirety to verify polymerase fidelity.

Luciferase reporter assays were performed by transiently transfecting HEK 293T cells respectively with 200 ng of pMIR-report-JMJD1A 3′UTR, pMIR-report-BACH1 3′UTR, pMIR-report-vector (control), together with 30 nM miR-155 Pre-miR™ miRNA Precursor Molecules (Cat#: PM12601, Ambion, USA), or Pre-miR™ miRNA Precursor Molecules-Negative Control #1 (Cat#: AM17110, Ambion, USA) and 200 ng of pCMV-Renilla (internal control) using Lipofectamine 2000 (Invitrogen) respectively. Firefly and Renilla luciferase activities were measured consecutively by using Dual Luciferase Assay (Cat#: E1910, Promega, USA) 48 hr after transfection. Firefly luciferase values have been normalized to Renilla, and the ratio of firefly/renilla was presented.

### *In situ* hybridization (ISH)

In situ detection of miR-155 was performed on 5 µm FFP tissue sections of NPC. Sections were prehybridized in hybridization solution (50% formamide, 5× SSC, 0.5 mg/mL yeast tRNA, 1× Denhardt's solution) for 30 minutes before hybridization. MiR-155 miRCURY LNA™ Detection probe (Cat#: 38537-05, Exiqon, Denmark) was hybridized to the sections for 1 hr at 25°C lower than predicted Tm of the probe. After posthybridization washes, in situ hybridization signals were detected using the tyramide signal amplification system (Perkin-Elmer) according to the manufacturer's instructions. Slides were mounted in ProLong Gold containing 4′,6-diamidino-2-phenylindole (DAPI; Invitrogen) and analyzed with an Olympus MVX10 microscope equipped with a charge-coupled device camera and Olympus CellP software.

### Immunohistochemistry

Primary antibodies against JMJD1A (1∶ 100 dilution, Ab75620, Abcam, USA) and BACH1 (1∶ 800 dilution, Ab54814, Abcam, USA) were used in this study. Briefly, tissue sections were de-waxed, incubated with hydrogen peroxide for 10 minutes, incubated in retrieval buffer solution for antigen recovery, blocked with normal serum for 10 minutes and incubated with a primary antibody for 60 minutes, followed by detection using a Catalyzed Signal Amplification Kit (DAKO, USA); signal was visualized using diaminobenzidine. Non-immune goat or rabbit serum was substituted for the primary antibody as a negative control. The immunohistochemistry results were evaluated and scored by a senior pathologist without knowledge of the clinicopathological outcomes of the patients.

A semiquantitative estimation was made by using a composite score obtained by adding the values of the staining intensity and the relative abundance of positive cells. The intensity was graded as 0 (no staining), 1 (weak staining), 2 (moderate staining) and 3 (strong staining). The abundance of the positive cells was graded from 0 to 3 (0, <5% positive cells; 1, 5–25%; 2, 26–50%; 3, >50%). A composite score greater than the median value was considered as high expression, and composite scores less than or equal to the median value were considered as low expression.

### Statistical analysis

Data was analyzed using SPSS12.0 software. The association between JMJD1A and BACH1 expression and clinicopathological parameters were assessed using a Chi-Square test. Kaplan-Meier analysis and log-rank tests were used to assess the survival rate and to compare the difference in survival curves. It was considered as significant differences when p<0.05.

## Supporting Information

Figure S1 **Comparison of the results of miRNAs microarray and qRT-PCR.** Comparison of miR155, miR146a and miR200c fold-changes by miRNAs microarray and qRT-PCR in the pair of TW03LMP1/TW03 (A) and the pair of TW03LMP2A/TW03 (B).
(TIF)

File S1 **The characteristics of the 1992 NPC staging system.**
(DOC)

Table S1 **The potential target genes of miR-155 predicted by at least three algorithms.**
(XLS)
