# Supplementary figures and images for: Upregulation of MiR-155 in Nasopharyngeal Carcinoma is Partly Driven by LMP1 and LMP2A and Downregulates a Negative Prognostic Marker JMJD1A
Source: PLoS One. 2011 Apr 26;6(4):e19137. doi: 10.1371/journal.pone.0019137 (PMC3082546; doi:10.1371/journal.pone.0019137)

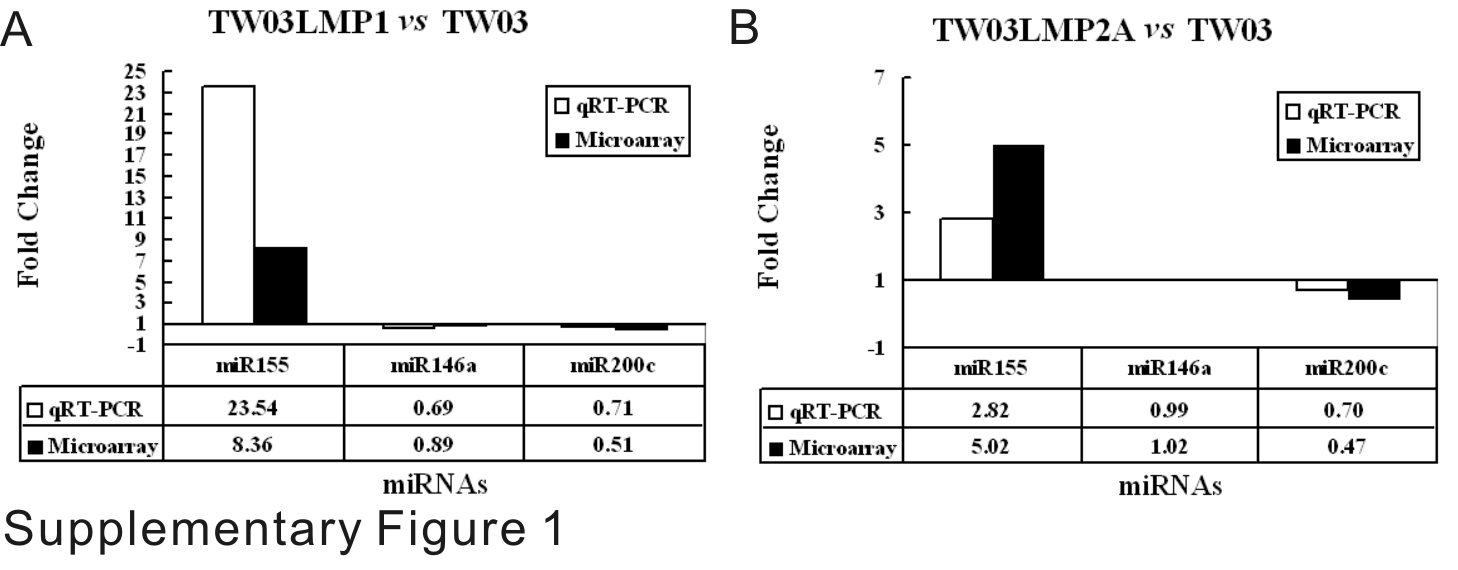

Supplement: Figure S1 — Comparison of the results of miRNAs microarray and qRT-PCR. Comparison of miR155, miR146a and miR200c fold-changes by miRNAs microarray and qRT-PCR in the pair of TW03LMP1/TW03 (A) and the pair of TW03LMP2A/TW03 (B). (TIF) [file pone.0019137.s001.tif]
