# Supplementary material for: Upregulation of MiR-155 in Nasopharyngeal Carcinoma is Partly Driven by LMP1 and LMP2A and Downregulates a Negative Prognostic Marker JMJD1A
Source: PLoS One. 2011 Apr 26;6(4):e19137. doi: 10.1371/journal.pone.0019137 (PMC3082546; doi:10.1371/journal.pone.0019137)
Supplement: File S1 — The characteristics of the 1992 NPC staging system. (DOC) [file pone.0019137.s002.doc]

# File S1. The characteristics of the 1992 NPC staging system

The staging system is characterized according to the following model: T, primary tumor: T1, limited to the nasopharynx; T2, involvement of the nasal cavity, oropharynx, soft palatine, anterior cervical vertebrae soft tissue, and parapharyngeal space extension before the SO line (the SO line is between the styloid process and the midpoint on the posterior edge of the great occipital foramen);T3, extension over the SO line, involvement of the anterior or posterior cranial nerves alone, the base of the skull, the pterygoprocess zone, and the pterygopalatine fossa; T4, involvement of both anterior and posterior cranial nerves, parabasal sinus, cavernous sinus, orbit, infratemporal fossa, and direct invasion of the first or second cervical vertebrae; N, regional lymph node involvement: N0, no enlarged lymph nodes; N1, greatest dimension of upper neck lymph node <4 cm, movable; N2, lower neck lymph node or greatest lymph node dimension between 4 and 7 cm; N3, supraclavicular lymph node, lymph node greatest dimension >7 cm, fixed, or skin infiltration (the border between the upper neck and the lower neck is the inferior margin of the cricoid cartilage); M, distant metastasis: M0, absence of distant metastasis; M1, presence of distant metastasis; staging: Stage I, T1N0M0; Stage II, T2N0-N1M0, T0-T2N1M0; Stage III, T3N0-N2M0, T0-T3N2M0; Stage IVa, T4N0-N3M0, T0-T4N3M0; Stage IVb, M1.
